# A Valine Mismatch at Position 129 of *MICA* Is an Independent Predictor of Cytomegalovirus Infection and Acute Kidney Rejection in Simultaneous Pancreas–Kidney Transplantation Recipients

**DOI:** 10.3390/ijms19092618

**Published:** 2018-09-04

**Authors:** Rafael Tomoya Michita, José Artur Bogo Chies, Sabine Schramm, Peter A. Horn, Falko M. Heinemann, Andreas Wunsch, Richard Viebahn, Peter Schenker, Vera Rebmann

**Affiliations:** 1 Institute for Transfusion Medicine, University Hospital Essen, University Duisburg-Essen, 45147 Essen, Germany; rafael.michita@gmail.com (R.T.M.); sabine.schramm3@uk-essen.de (S.S.); peter.horn@uk-essen.de (P.A.H.); falko.heinemann@uk-essen.de (F.M.H.); 2 Post-Graduation Program in Genetics and Molecular Biology, Genetics Department, Universidade Federal do Rio Grande do Sul (UFRGS), Porto Alegre 91501-970, Brazil; jabchies@terra.com.br; 3 Department of Surgery, University Hospital Knappschaftskrankenhaus Bochum, Ruhr-University Bochum, 44892 Bochum, Germany; andreas.wunsch@kk-bochum.de (A.W.); richard.viebahn@kk-bochum.de (R.V.); peter.schenker@ruhr-uni-bochum.de (P.S.)

**Keywords:** simultaneous pancreas–kidney transplantation, Val129Met, MICA, cytomegalovirus, acute and latent cytomegalovirus infection, sMICA, dimorphism, genetic predisposition

## Abstract

The polymorphic major histocompatibility complex class I chain-related molecule A (MICA) and its soluble form (sMICA) interact with activating receptor natural-killer group 2 member D (NKG2D) on natural-killer (NK) and T cells, thereby modifying immune responses to transplantation and infectious agents (e.g., cytomegalovirus). Two single-nucleotide polymorphisms (SNPs), rs2596538GA in the *MICA* promoter and rs1051792AG in the coding region (*MICA*-129Val/Met), influence MICA expression or binding to NKG2D, with MICA-129Met molecules showing higher receptor affinity. To investigate the impact of these SNPs on the occurrence of cytomegalovirus infection or acute rejection (AR) in individuals who underwent simultaneous pancreas–kidney transplantation (SPKT), 50 recipient-donor pairs were genotyped, and sMICA levels were measured during the first year post-transplantation. Recipients with a Val-mismatch (recipient Met/Met and donor Val/Met or Val/Val) showed shorter cytomegalovirus infection-free and shorter kidney AR-free survival. Additionally, Val mismatch was an independent predictor of cytomegalovirus infection and kidney AR in the first year post-transplantation. Interestingly, sMICA levels were lower in rs2596538AA and MICA129Met/Met-homozygous recipients. These results provide further evidence that genetic variants of *MICA* influence sMICA levels, and that Val mismatch at position 129 increases cytomegalovirus infection and kidney AR risk during the first year post-SPKT.

## 1. Introduction

Simultaneous pancreas and kidney transplantation (SPKT) is the most effective treatment for diabetes mellitus type 1 patients with end-stage renal disease. Graft survival following SPKT has improved over the previous decade due to advances in immunosuppressive regimens: antithymocyte globulin and basiliximab induction therapies are used by the majority of transplant centers, with the former associated with lower rejection rates during the first year post-transplantation [1].

Despite the overall improvement in SPKT, acute rejection (AR) remains the major challenge in solid-organ transplantation, as it has a negative effect on the allograft function and may trigger chronic rejection. Notably, the occurrence of AR in SPKT is partially attributed to donor type, as the majority of pancreas and kidney donors are deceased (cardiac-dead and brain-dead donors). Consequently, allografts from such donors are more strongly affected by ischemia-reperfusion injury capable of causing delayed graft function and/or ultimate rejection.

Another complication influencing graft survival is human cytomegalovirus infection, which frequently occurs after transplantation and leads to severe complications. In solid-organ transplantation, the main risk factors associated with cytomegalovirus infection are serological mismatch between recipient and donor (recipient is cytomegalovirus-negative and donor is cytomegalovirus-positive; cytomegalovirus R−/D+), high doses of methylprednisolone, and T cell-depletion therapy [2]. In immunocompetent hosts, cytomegalovirus infection is usually asymptomatic; however, in immunosuppressed transplantation recipients, the infection manifests as a systemic syndrome that affects multiple organs. Importantly, cytomegalovirus infection increases the risk of opportunistic infections, allograft dysfunction, and the overall cost of transplantation.

The major histocompatibility complex (MHC) class I chain-related molecule A (*MICA*) encodes a stress-induced protein located within MHC loci and is close to the human leukocyte antigen (*HLA*)-*B* gene [3]. *MICA* is a highly polymorphic non-MHC gene that includes 107 alleles encoding 82 proteins, according to Immuno Polymorphism Database [4]. MICA proteins exist as membrane-bound and soluble molecules (sMICA), each with distinct biological properties. Unlike classic MHC class I molecules, MICA is not involved in antigen presentation, but rather acts as a ligand for natural-killer (NK) group 2-member D (NKG2D), an activating C-type lectin-like receptor. The constitutive expression of MICA is restricted to few cell types, including endothelial cells, dendritic cells, fibroblasts, and epithelial cells (reviewed in Reference [5]). MICA upregulation in tissues indicates virus-induced stress or injury, malign transformation, or ischemia-reperfusion injury of allografts in the transplantation setting [6]. Because MICA expression functions as a costimulatory signal for CD8+ T cells and triggers cytotoxic and cytokine immune responses by NK effectors, its expression and potential for stress-induced upregulation represent an additional boundary between tolerance and rejection in allogeneic situations such as transplantation.

*MICA* single-nucleotide polymorphisms (SNPs) influence expression patterns of the gene. Specifically, rs2596538G/A located in the promoter region at position –2778 from exon 1 influences sMICA levels by modifying the affinity to transcription factor specificity protein-1. The variant rs2596538G is associated with higher sMICA expression levels, especially in patients with hepatitis C virus-induced hepatocellular carcinoma [7]. SNP rs1051792, also known as *MICA*-129Val/Met, is characterized by a substitution of guanine with adenine at position 454 in exon 3, which results in a nonsynonymous mutation of valine to methionine at codon 129. *MICA*-129Val/Met not only affects sMICA levels, but also its affinity to the NKG2D receptor expressed on CD8+ T cells, γδ T cells, and NK cells. MICA-129Met proteins bind soluble NKG2D with a 10- to 50-fold higher affinity than MICA-129Val proteins [5,8]. Additionally, MICA-129Met proteins are expressed at low levels in the cell membrane due to intracellular retention, and are highly susceptible to membrane shedding [9].

The clinical relevance of *MICA*-129Val/Met has been highlighted by diverse association studies, including those in patients with autoimmune disorders, malignancies, and infections [3,10]. Recent studies on hematopoietic stem-cell transplantation have produced conflicting results, with different MICA-129 genotypes being associated with different outcomes [11,12,13,14,15]. The effects of both functionally relevant SNPs (rs2596538 and *MICA*-129Val/Met) on the incidence of cytomegalovirus infection and AR after SPKT remain unknown. Therefore, we investigated the impact of these SNPs on the occurrence of cytomegalovirus infection or AR in SPKT by genotyping 50 recipient-donor pairs and defining their sMICA levels during the first year post-transplantation.

## 2. Results

Clinical and demographic characteristics of SPKT recipients with or without at least one AR event are provided in Table 1. AR was observed in 19 (38%) individuals with SPKT and at different frequencies in kidney (84.2%) and pancreas (21.1%) allografts. Only one patient exhibited graft rejection of both. The median age, biochemical parameters, cold ischemia time of the graft, and the number of human leukocyte antigen (*HLA)-A*, *-B*, and *-DR* mismatches between AR and functional/stable graft recipients were similar. Before transplantation, anti-MICA antibodies were more frequently detected in patients in the AR group (26.3% vs. 3.4%; *p* = 0.030) as compared with those without AR. After transplantation, the frequencies of anti-MICA antibody detection were similar in both groups.

Cytomegalovirus-positive recipients were more prevalent among those with stable grafts than with AR allografts (61.3 vs. 26.3; *p* = 0.016), and the frequency of patients receiving cytomegalovirus-positive grafts was higher in the AR group (42.1 vs. 16.1, *p* = 0.042). After a one-year follow-up, active infection was observed with similar frequencies in both groups (AR: 15.8% vs. stable grafts: 12.9%; *p* > 0.05).

**Figure 1:**
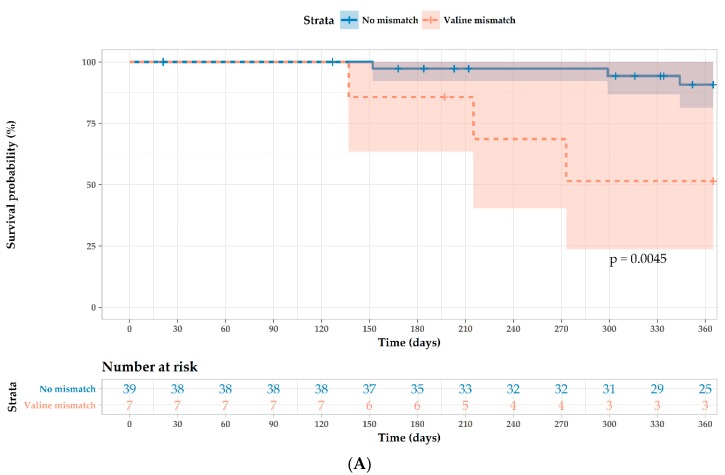

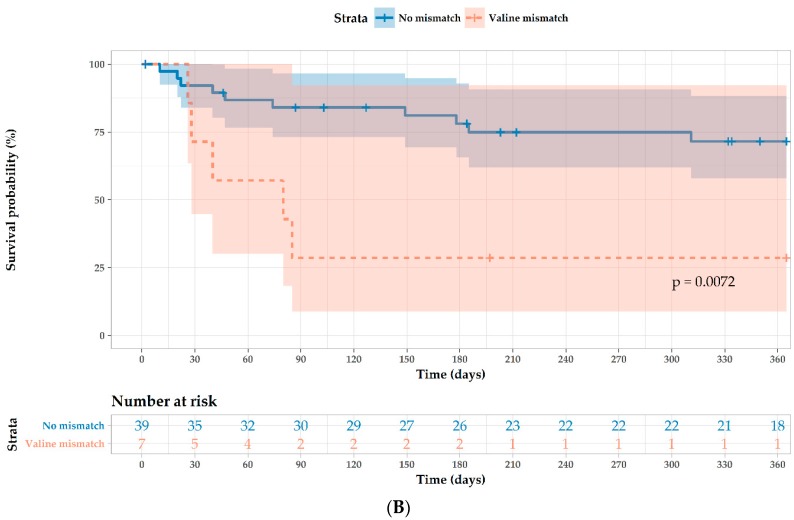
Impact of MICA-129Val/Met on survival following simultaneous pancreas and kidney transplantation. (**A**) Effect of MICA-129-Val mismatch (recipient Met/Met and donor Met/Val or Val/Val) in patients with cytomegalovirus infection at one year post-transplantation. (**B**) Effect of MICA-129-Val mismatch in patients with kidney acute rejection in one year post-transplantation.

We then evaluated the impact of *MICA*-129Val/Met mismatches between recipient and donor pairs. Kaplan–Meier curves indicated that *MICA*-129 mismatch status (recipient Met/Met and donor Met/Val or Val/Val) conferred shorter cytomegalovirus-infection-free survival (*p* = 0.004; Figure 1A) and kidney-rejection-free survival rates (*p* = 0.007; Figure 1B) during the first year post-SPKT. Multivariate Cox regression analysis indicated that *MICA*-129 mismatch represented an independent prognostic risk factor for cytomegalovirus infection (*p* = 0.049; hazard ratio (HR): 5.32; 95% confidence interval (CI): 1.00–28.1; Table 2) and kidney AR (*p* = 0.006; HR: 6.04; 95% CI: 1.68–21.7, Table 3) during the first year post-transplantation.

Additionally, in the first year post-transplantation: SPK patient, pancreas-, and kidney-graft survival rates were 96%, 84%, and 88%, respectively. In recipients without valine mismatch: SPK patient, pancreas-, and kidney-graft survival rates were 97.5%, 90%, and 92.5%. In the valine mismatch group: SPK patient, pancreas-, and kidney-graft survival rates were slightly worse, but this difference did not reach statistical significance (90%, 70%, and 60%, respectively).

Evaluation of *MICA*-129Val/Met genotypes based on plasma sMICA levels from SPKT recipients showed that in all measurements, Met/Met homozygotes had the lowest plasma sMICA levels (below the detection limit) relative to those in patients with Val/Val and Val/Met genotypes (*p* = 0.005, Table 4). Evaluation of SNP rs2596538G/A in the *MICA*-promoter region revealed that for all measurements, rs2596538AA homozygotes had the lowest median sMICA levels, with these differences being more pronounced when AA homozygotes were compared with rs2596538G-allele carriers [GG + GA = 0.73 (range: 0.54–1.05) vs. AA < 0.02; *p* = 0.025]. Furthermore, additional sMICA measurements from voluntary blood donors confirmed that *MICA*-129Met/Met and rs2596538AA genotypes were associated with the lowest sMICA levels (*p* < 0.001).

Analysis of linkage disequilibrium (LD) between *MICA* variants in recipients (D’ = 0.61, r^2^ = 0.37) and recipients plus donors (D’ = 0.64, r^2^ = 0.40) suggested only recombination and low incidence of LD. Interestingly, 89.9% (8/9) of *MICA*-129Met/Met homozygotes carried at least one rs2596538A allele, with 55.6% (5/8) being homozygous for this allele (rs2596538AA). Furthermore, 96.3% (26/27) of *MICA*-129Val/Val homozygotes carried at least one rs2596538G allele, with 77.8% (21/27) being homozygous for this allele (rs2596538GG). Similar to SPKT recipients, all Met/Met homozygotes carried at least one rs2596538A allele, and 57.1% of them (4/7) were also rs2596538AA homozygous, whereas all Val/Val homozygotes harbored at least one rs2596538G allele, and 70.0% (14/20) were also GG homozygous.

## 3. Discussion

Studies evaluating *MICA*-129 mismatch in solid-organ transplantation are scarce, as *MICA* typing is seldom reported, except for the detection of anti-MICA antibodies, which affect allograft survival [16,17]. In the present study, we report that the direction and type of *MICA* mismatches, rather than their number, influence SPKT outcomes. Specifically, we observed for the first time that *MICA*-129Val mismatch (donor *MICA*-129Val carrier and recipient *MICA*-129Met homozygote) is an independent predictor of cytomegalovirus infection and kidney AR in the first year post-SPKT. The functional implications of variability of MICA-129 molecules that bind to NKG2D with low (Val) or high (Met) affinity have been reported [12]. In hematopoietic stem-cell transplantation studies, *MICA* has gained special attention due to the functional impact of MICA-129Val/Met dimorphism. However, despite extensive investigations, the question of whether *MICA* mismatches increase or decrease the risk of graft-versus-host disease or overall patient survival remains a matter of debate [11,12,13,14,15].

The increased risk of AR in recipients harboring *MICA*-129Met/Met can be explained by the low level of sMICA present in AR patients, which could cause insufficient host immune suppression due to the downregulation of surface NKG2D expression on NK and CD8+ T cells. We also suggest that the expression of membrane MICA-129 valine molecules on the allograft tissue has potential for stimulating an immune response in *MICA*-129Met/Met recipients and leading to worse clinical outcomes.

Consistent with our observations, decreased expression of membrane-bound and sMICA molecules is associated with the *MICA*-129Met allele [12]. However, it should be noted that in our study, Met/Met homozygotes were likely to be rs2596538AA homozygous, and the latter genotype is associated with lower sMICA levels. Moreover, the rs2596538G allele influences specificity protein-1 transcription-factor binding, and thus is associated with higher sMICA levels than the rs2596538A allele [7].

In heart and kidney transplantation patients, consistent sMICA expression is associated with improved graft tolerance [18,19]. In our SPKT study, *MICA*-129Val/rs2596538G carriers exhibited the highest sMICA levels, whereas *MICA*-129Met/Met and rs2596538AA homozygotes had the lowest sMICA levels. Concomitant with low sMICA levels, *MICA*-129Met/Met carriers are exposed to MICA-129Val proteins expressed on donor allograft cells (Val mismatches), with the expression levels of these proteins correlating with NKG2D-mediated downstream signaling in NK and CD8+ T cells that promotes secretion of tissue-injury related mediators. Additionally, overexpression of *MICA*-129Met proteins leads to NKG2D internalization, resulting in impaired functions of both NK and CD8+ T cells [10,12]. Therefore, the evaluation of recipient-donor *MICA*-129Val/Met genotypes might provide reliable markers for patients that are at higher AR risk based on the mismatch type.

Currently, few studies evaluated the impact of MICA genetic variation on the clinical outcome of immunologic disorders and transplantation (reviewed in Reference [10]). An exception includes a study evaluating the variation in regulatory regions of the gene, such as the rs2596542 and rs2596538 SNPs [7]. The investigation and impact of *MICA* alleles in distinct outcomes are even more limited since *MICA* gene is highly polymorphic (>100 alleles). Consequently, a comprehensive evaluation requires high-throughput methodologies. Noteworthy, MICA Val129Met genotyping allows differentiation of the most frequent alleles in two groups.

It has been shown that genotyping of *MICA*-129Val/Met allows classification of *MICA* alleles by low (Val) or high (Met) affinity of the protein product to NKG2D [5]. Therefore, depending on the population under investigation, this might provide a rough estimate of segregated *MICA* alleles. We were unable to confirm donor–recipient *MICA*–allele typing in SPKT patients. However, *MICA*-129Val is present in the MICA*008 allele, which is most frequently identified in Caucasians, and is currently under intense investigation [5].

Interestingly, the product of MICA*008 lacks the cytoplasmic domain due to a microsatellite SNP (A5.1). However, this allele still expresses a protein on the membrane through a dynamic mechanism involving glycosylphosphatidylinositol anchoring. Because the latter is a slow process, unbound MICA proteins can be released as nanovesicles and soluble proteins, indicating that mechanisms other than those currently explored (i.e., SNPs) impact *MICA* expression patterns [10,20]. Additionally, it is possible that MICA*008- and *MICA*-129Val-expression patterns overlap.

In the specific context of cytomegalovirus infection, cytomegalovirus eludes host immune system by targeting and downregulating NKG2D ligands at multiple checkpoints, including those involved in protein sequestration, mRNA degradation, translational repression, protein degradation, in the allele-specific manner [21]. The MICA*008 allele illustrates this specificity as it represents an “escape variant” resistant to most cytomegalovirus viral glycoproteins, except for the recently identified US9 protein. Therefore, MICA*008 carriers are less prone to cytomegalovirus infection than individuals with full-length *MICA* alleles [22,23]. A higher risk of cytomegalovirus reactivation is associated with A5.1 homozygosity (which includes the MICA*008 allele) in HIV-immunodeficient patients [24]. It should be noted that HIV-1 Nef protein downregulates *MICA* expression, and this circumstance needs to be taken into account in coinfected and immunosuppressed patients [25].

Consequently, we suggest that susceptibility to cytomegalovirus infection in SPKT represents a cumulative effect of recipient–donor *MICA*-129Val/Met genotypes, with equivalent effects of serological mismatches, immunosuppressive regimens, and lymphocyte-depletion therapy. In this study, we observed that Val-mismatch recipients were at higher risk of cytomegalovirus infection post-SPKT. The fact that Met/Met homozygotes lack the “escape variant” and likely carry a 2596538A allele makes them susceptible to cytomegalovirus-specific immune evasion because *MICA* expression becomes downregulated in favor of viral replication [21,26]. However, additional studies are required to clarify the mechanisms associated with the susceptibility of Val-mismatch recipients to cytomegalovirus-infection risk, as well as the impact of donor-allograft genotypes and different cytomegalovirus strains. Although we focused our attention on cytomegalovirus infection, our observations are also relevant for assessing the risk of other opportunistic infections, especially those caused by other herpesviruses that adopt multiple means to evade immune response, including *MICA* downregulation [27]. It is noteworthy that *MICA* Val129Met SNP influence on cytomegalovirus infection and AR could be specific for SPKT since the current cohort under investigation differs in the clinical practice/management and characteristics of other types of solid organ transplantation. Consequently, it makes SPKT and kidney only transplantation not comparable, although the functional relevance of *MICA* Val129Met SNP may overlap.

In summary, for the first time, we evaluated *MICA*-129Val/Met and rs2596538G/A genetic variations in SPKT recipients. We demonstrated that *MICA*-129Val mismatch is an effective predictor of AR risk and cytomegalovirus infection in the first year post-SPKT. Additionally, *MICA*-129Val/Met genotypes affect overall sMICA levels in individuals with SPKT, and, more importantly, rs2596538G/A SNP in the *MICA*-promoter region also affects sMICA levels. These findings indicated that evaluation of *MICA*-129-mismatch in individuals with SPKT might help to stratify them by the probable risks of developing AR and contracting cytomegalovirus infection. Furthermore, a simultaneous determination of rs2596538G/A genotype in the *MICA*-promoter region might provide a better understanding of the mechanisms associated with sMICA expression, which will be beneficial in the context of allotransplantation.

## 4. Materials and Methods

### 4.1. Study Population and Outcome Parameters

A total of 50 diabetes mellitus type 1 patients undergoing SPKT were recruited from 2012 to 2016 at Universitätsklinikum Knappschaftskrankenhaus (Bochum, Germany). Induction immunosuppression was employed in all patients. Most patients received thymoglobulin (Sanofi, Frankfurt, Germany) induction in daily doses of 1.5 mg/kg body weight for 3 days. Maintenance immunosuppression consisted of tacrolimus, mycophenolic acid, and prednisolone in most cases.

Serum creatinine, blood-glucose values, and serum-lipase levels were used to detect allograft dysfunction. When rejection was suspected, a biopsy of the kidney graft was performed primarily and graded according to the BANFF classification [28]. A biopsy of the pancreas graft was performed only when isolated pancreas graft rejection was suspected. AR episodes were treated with methylprednisolone initially for 3 consecutive days, and, if resistant, with thymoglobulin. Antibody-mediated rejections were treated with thymoglobulin and/or plasmapheresis and intravenous immune globulin.

All patients received cytomegalovirus prophylaxis with ganciclovir, followed by valganciclovir for at least 3 months postoperatively. In individuals with presumed high-risk sensitivity to cytomegalovirus (D+/R−; D+/R+), cytomegalovirus prophylaxis was prolonged to 6 months. Surveillance for cytomegalovirus infection was performed by PCR (Cobas 4800; Roche Diagnostics, Mannheim, Germany) weekly during the first month following SPKT and monthly thereafter until the end of the first year or in cases of clinical suspicion. Any level of detection was considered positive. Cytomegalovirus disease was defined as cytomegalovirus infection with at least one of the symptoms: newly occurred malaise, fever > 38 °C, leukopenia and thrombocytopenia, elevation of hepatic transaminases to more than twice the standard values, or graft dysfunction. Treatment of cytomegalovirus infection was performed using intravenous ganciclovir adjusted to the calculated glomerular-filtration rate, followed by 3 additional months of prophylactic valganciclovir treatment.

Recipients and deceased-donor grafts were typed for *HLA-A*, *-B*, and *-DR*. The recipients were followed-up with over a 1-year period after SPKT and evaluated for the occurrence of graft failure, cytomegalovirus infection, and AR. Demographic and clinical data were collected before and after SPKT and summarized according to cytomegalovirus status and AR outcomes (Table 1). Voluntary blood donors (*n* = 44) were included in the study in order to evaluate the effect of *MICA*-129Val/Met and rs2596538G/A genotypes on sMICA levels. The protocols used in this study were approved by the local ethics board of the Faculty of Medicine, Ruhr-University of Bochum (No. 12-4380; date: 20 September 2012) and were in agreement with the Declaration of Helsinki. Before inclusion in the study, all participants provided written informed consent.

### 4.2. MIC-A 454G/A and rs2596538A/G Genotyping

Genomic DNA was obtained using a QIAmp DNA mini kit (Qiagen, Hilden, Germany) according to manufacturer’s instructions from whole-blood samples collected in ethylenediaminetetraacetic acid. Genotypes associated with SNP rs1051792 involving an A > G substitution at position 454 in exon 3 of the *MICA* gene were determined by nested PCR, followed by RsaI (New England Biolabs, Ipswich, MA, USA) restriction digestion (PCR conditions are described in Table S1). Electrophoresis was performed on a 2.5% agarose gel at 140 V for 20 min in order to visualize digestion patterns and determine the presence of MIC-A 454G/A genotypes associated with the following nonsynonymous substitutions: 454GG = Val/Val (106 and 21 bp (base pair)); 454GA = Val/Met (127, 106, and 21 bp), and 454AA = Met/Met (127 bp).

Genotyping of rs2596538A/G was performed using a PCR-restriction fragment length polymorphism technique described in Table S2. All PCR amplifications were evaluated by 1% agarose gel electrophoresis and AluI (New England Biolabs) restriction digest reactions according to manufacturer instructions. The amplified sequences had a total of 339 bp and contained 4 AluI-recognition sites, yielding the following fragments after digestion: 139, 92, 78, 23, and 7 bp. The presence of the A allele led to the digestion of the 170 bp fragment resulting in the segments of 78 and 92 bp in length. Electrophoresis on a 3.5% agarose gel was used to visualize the digestion patterns and determine the following *MICA* rs2596538A/G genotypes: AA (139, 92, 78, 23, and 7 bp), AG (170, 139, 92, 78, 23, and 7 bp), and GG (170, 139, 23, and 7 bp). Samples were processed in batches, including all known genotypes as additional positive controls. At the end, 10% of samples were randomly selected and retested; 100% reproducibility was obtained for both methods.

### 4.3. sMICA Measurement

Determination of sMICA levels was performed as previously described [29]. Briefly, sMICA molecules were captured using an AMOI antibody (BAMOMAB, Munich, Germany) at a final concentration of 25 ng/mL. Detection of bound sMICA molecules was performed using a biotin-labeled polyclonal goat anti-human MICA antibody (R and D Systems GmbH, Wiesbaden-Nordenstadt, Germany) diluted to 400 ng/mL in phosphate-buffered saline containing 1% bovine serum albumin and 2% inactivated goat serum. Detection of bound antibodies was performed by streptavidin conjugated with horseradish peroxidase (R and D Systems GmbH) diluted 1:200 in phosphate-buffered saline containing 1% bovine serum albumin. 3,3′,5,5′-tetramethylbenzidine (Sigma-Aldrich Chemie GmbH, Munich, Germany) served as substrate solution, and absorbance was measured at 450 nm (Biotek Instruments, Winooski, VT, USA). Recombinant MICA protein fused with the Fc portion of human IgG (R and D Systems GmbH) was used as standard. The detection limit of sMICA was 20.4 pg/mL or 0.024 ng/mL, with detection limits calculated according to DIN 32645 standard. Intra-assay coefficients of variation were 4.1%, and interassay coefficients of variation were 14.3%.

### 4.4. Statistical Analysis

Asymmetric distributions were described by the median (25–75 percentiles). Continuous and categorical variables were compared using the Mann–Whitney U or Kruskal–Wallis test and chi-squared tests, as appropriate. The SPKT outcomes evaluated included AR, graft function, and cytomegalovirus infection in the first year post-SPKT. Survival in groups with allograft AR and cytomegalovirus infection was estimated by Kaplan–Meier curves and evaluated through the log-rank test implemented in the R package survminer (v0.4.0; https://www.r-project.org/). Multivariate Cox regression according to proportional hazards assumption was used to assess the risk of allograft AR and cytomegalovirus infection. Covariates were entered in the multivariate analysis based on conceptual evaluation of the literature or/and by means of a statistical threshold (association of the covariate with the outcome and with the study factor at *p* ≤ 0.20). LD was tested by D’ and r^2^ statistics was calculated by using Haploview software (https://www.broadinstitute.org/haploview/haploview). All remaining analyses were performed in SPSS for Windows (v18.0; SPSS Inc., Chicago, IL, USA). Effects were considered statistically if *p* < 0.05.

## Figures and Tables

**Table 1:** Clinical and demographic characteristics of SPKT patients stratified by the presence of AR in the first year post–transplantation.

Variables	AR (*n* = 19)	No AR (*n* = 31)	*p*
Age (median: 25–75%)	50 (35.0–56.5)	43.0 (34.5–53.0)	0.289
Recipient gender (male/female)	10/9	19/12	0.547
Donor gender (male/female)	11/8	14/17	0.382
BMI (median: 25–75%)	24.2 (21.8–26.43)	25.0 (20.8–27.8)	0.818
Urea, mg/dL (median: 25–75%)	18.6 (16.1–27.2)	18.5 (15.4–20.5)	0.431
Creatinine, mg/dL (median: 25–75%)	1.36 (1.09–1.61)	1.30 (1.02–1.55)	0.516
Glucose, mg/dL (median: 25–75%)	104 (90.0–119.0)	97.5 (88.0–116.0)	0.382
HbA1C, % (median: 25–75%)	5.85 (5.33–6.70)	5.70 (5.3–6.4)	0.693
Kidney graft cold ischemia time, min (±SD)	816.7 (158.4)	798.4 (173.9)	0.772
Pancreas graft cold ischemia time, min (±SD)	677.4 (133.4)	682.6 (137.2)	0.697
*HLA-A* and *-B* mismatches (median: 25%–75%)	3.0 (3.0–4.0)	3.0 (2.0–3.0)	0.267
*HLA–DR* mismatch (median: 25–75%)	2.0 (1.5–2.0)	2.0 (1.0–2.0)	0.225
Anti–MICA pretransplantation	5 (26.3)	1 (3.4)	**0.030**
Anti–MICA post-transplantation	2 (11.1)	4 (14.8)	1.000
Anti–MHC I post-transplantation	5 (27.8)	5 (18.5)	0.489
Anti–MHC II post-transplantation	4 (22.3)	6 (22.2)	1.000
Cytomegalovirus D+	11 (57.9)	11 (35.5)	0.121
Cytomegalovirus R+	5 (26.3)	19 (61.3)	**0.016**
Cytomegalovirus, R−/D+	8 (42.1)	5 (16.1)	**0.042**
Cytomegalovirus infection, first-year post-transplantation	3 (15.8)	4 (12.9)	1.000
Pancreas and kidney graft AR	1 (5.2)	ND	
Pancreas graft AR	4 (21.1)	ND	
Kidney graft AR	16 (84.2)	ND	
Immunosuppressive drugs (*n* (%))			
ATG	19 (100)	31 (100)	
Steroids	19 (100)	31 (100)	
Tacrolimus	18 (94.7)	30 (96.8)	
Mycophenolic acid	19 (100)	30 (96.8)	
Cyclosporine A	1 (5.3)	1 (3.2)	
Azathioprine	ND	1 (3.2)	
Simulect	ND	1 (3.2)	
Rituximab	ND	1 (3.2)	

Bold *p* values indicate statistically different parameters. AR = acute rejection; ATG = antithymocyte globulin; BMI = body-mass index; D = donor; *HLA* = human leukocyte antigen; *MICA* = MHC class I-related sequence A; R = recipient; SD = standard deviation; SPKT = simultaneous pancreas and kidney transplantation. ND = no data available.

**Table 2:** Univariate and multivariate Cox regression analysis of individual covariates and their impact on the risk of cytomegalovirus infection.

Variables	Univariate Analysis		Multivariate Analysis	
	HR (95% CI)	*p*	HR (95% CI)	*p*
Univariate analysis (*n* = 7)				
Valine mismatch ^1^	7.37 (1.47–36.9)	**0.015**	5.32 (1.0–28.1)	**0.049**
MICA antibodies pretransplantation ^2^	0.04 (0.00–1381.6)	0.546		
*HLA* mismatch ^3^				
4/6	1.39 (0.12–15.3)	0.790		
5/6	0.80 (0.07–8.76)	0.851		
6/6	1.74 (0.16–19.2)	0.651		
Cytomegalovirus R−/D+ ^4^	4.29 (0.96–19.6)	0.057	4.89 (0.86–27.9)	0.074
Kidney graft ischemia time	1.00 (1.00–1.00)	0.860		
Kidney graft AR	1.61 (0.36–7.20)	0.532		

Reference categories: ^1^ No mismatch. ^2^ No donor-specific antibodies. ^3^ 3/6 mismatch number (*HLA*-*A*, -*B*, and -*DR*). ^4^ Recipient cytomegalovirus+. CI = confidence interval; HR = hazard ratio. Bold values indicate statistically significant effects.

**Table 3:** Univariate and multivariate Cox regression analyses of predictor variables in SPKT patients with acute kidney rejection.

Variables	Univariate Analysis		Multivariate Analysis	
	HR (95% CI)	*p*	HR (95% CI)	*p*
Univariate analysis (*n* = 16)				
Valine mismatch ^1^	4.00 (1.4–12.0)	**0.012**	6.04 (1.68–21.7)	**0.006**
MICA antibodies pretransplantation ^2^	2.80 (0.89–8.7)	0.077	3.40 (0.84–13.8)	0.086
*HLA* mismatch ^3^				
4/6	2.91 (0.32–26.0)	0.340	3.65 (0.39–34.0)	0.256
5/6	4.02 (0.49–32.8)	0.193	5.34 (0.64–44.7)	0.122
6/6	4.03 (0.45–36.1)	0.212	3.11 (0.32–30.6)	0.330
*HLA-DR* mismatch ^4^	1.82 (0.58–5.64)	0.300		
Gender mismatch ^5^	1.47 (0.51–4.20)	0.480		
Cytomegalovirus infection ^6^	1.40 (0.40–4.90)	0.590		
Kidney graft ischemia time	1.00 (1.00–1.00)	0.860		

Acute kidney rejection (*n* = 16). Reference categories: ^1^ No mismatch. ^2^ No donor-specific antibodies. ^3^ 3/6 mismatch number (*HLA-A*, -*B*, and -*DR*). ^4^ *HLA*-*DR* mismatch number (*n* = 0 or 1). ^5^ Female recipient and male donor. ^6^ No infection in the first year post-transplantation. Bold values indicate statistically significant effects.

**Table 4:** Effect of Val129Met substitution and rs2596538GA on sMICA levels in SPKT recipients.

Measurements, Days after Transplantation (± SD)	Val/Val	Val/Met	Met/Met	*p* *
Val129Met				
1st, 0.58 (0.90) ^1^	0.87 (0.75–1.44)	1.22 (0.60–1.75)	<0.02	0.265
2nd, 98.5 (46.0) ^2^	0.42 (0.35–0.90)	0.55 (0.53–0.88)	<0.02	**0.007**
3rd, 238.9 (72.5) ^3^	0.66 (0.40–0.75)	0.50 (0.45–1.08)	<0.02	**0.035**
Median values at one year post-transplantation ^4^	0.69 (0.49–0.87)	1.04 (0.71–1.19)	<0.02	**0.005**
Healthy individuals **	1.16 (0.88–1.54)	0.55 (0.44–0.62)	0.00 (0.00–0.04)	**<0.001**
rs2596538GA	**GG**	**GA**	**AA**	
First, 0.58 (0.9) ^1^	0.87 (0.65–1.59)	0.87 (0.81–1.64)	<0.02	0.241
Second, 98.5 (46.0) ^2^	0.49 (0.42–0.76)	0.55 (0.45–0.72)	0.00 (0.00–0.45)	0.103
Third, 238.9 (72.5) ^3^	0.58 (0.32–1.17)	0.63 (0.47–0.89)	<0.02	**0.036**
Median values at one year post-transplantation ^4^	0.76 (0.60–1.17)	0.71 (0.47–0.97)	<0.02	0.075
Healthy individuals **	1.37 (0.91–1.55)	0.58 (0.43–0.83)	0.00 (0.00–0.06)	**<0.001**

sMICA values are presented as medians (25%–75%). Bold values indicate statistically significant effects. ^1^ First measurement: Val129Met Val/Val (*n* = 6), Val/Met (*n* = 6), and Met/Met (*n* = 1); rs2596528 GG (*n* = 7), GA (*n* = 5), and AA (*n* = 1). ^2^ Second measurement: Val129Met Val/Val (*n* = 8), Val/Met (*n* = 7), and Met/Met (*n* = 4); rs2596538 GG (*n* = 7), GA (*n* = 7), and AA (*n* = 5). ^3^ Third measurement: Val129Met Val/Val (*n* = 6), Val/Met (*n* = 5), and Met/Met (*n* = 3); rs2596538 GG (*n* = 4), GA (*n* = 7), and AA (*n* = 3). ^4^ Median values at one year post-transplantation: Val129Met Val/Val (*n* = 10), Val/Met (*n* = 7), and Met/Met (*n* = 4); rs2596538 GG (*n* = 8), GA (*n* = 8), and AA (*n* = 5). sMICA-detection limit: >0.02 pg/mL. * Kruskal–Wallis test. ** Healthy voluntary individuals: Val/Val (*n* = 20), Val/Met (*n* = 11), and Met/Met (*n* = 7); rs2596358, GG (*n* = 16), GA (*n* = 23), and AA (*n* = 5). sMICA = soluble MICA.

## Supplementary Materials

The following are available online at http://www.mdpi.com/1422-0067/19/9/2618/s1.

## Abbreviations

AR	Acute rejection
bp	base pair
D	Donor
HIV	Human immunodeficiency virus
HLA	Human leukocyte antigen
LD	Linkage disequilibrium
Met	Methionine
MHC	Major histocompatibility complex
MICA	Major histocompatibility complex class I chain-related molecule A
NK	Natural killer
NKG2D	Natural-killer group 2 member D
PBS	Phosphate buffered saline
R	Recipient
SD	Standard deviation
sMICA	soluble MICA
SNP	Single nucleotide polymorphism
SPKT	Simultaneous pancreas-kidney transplantation
Val	Valine

## References

[1] Bazerbachi F., Selzner M., Boehnert M.U., Marquez M.A., Norgate A., McGilvray I.D., Schiff J., Cattral M.S. (2011). Thymoglobulin versus basiliximab induction therapy for simultaneous kidney-pancreas transplantation: Impact on rejection, graft function, and long-term outcome. Transplantation.

[2] Razonable R.R., Rivero A., Rodriguez A., Wilson J., Daniels J., Jenkins G., Larson T., Hellinger W.C., Spivey J.R., Paya C.V. (2001). Allograft rejection predicts the occurrence of late-onset cytomegalovirus (CMV) disease among CMV-mismatched solid organ transplant patients receiving prophylaxis with oral ganciclovir. J. Infect. Dis..

[3] Baranwal A.K., Mehra N.K. (2017). Major Histocompatibility complex class i chain-related A (MICA) molecules: Relevance in solid organ transplantation. Front. Immunol..

[4] Robinson J., Halliwell J.A., Hayhurst J.D., Flicek P., Parham P., Marsh S.G.E. (2015). The IPD and IMGT/HLA database: Allele variant databases. Nucleic Acids Res..

[5] Risti M., Bicalho M.D.G. (2017). MICA and NKG2D: Is There an Impact on Kidney Transplant Outcome?. Front. Immunol..

[6] Luo L., Lu J., Wei L., Long D., Guo J.Y., Shan J., Li F.S., Lu P.Y., Li P.Y., Feng L. (2010). The role of HIF-1 in up-regulating MICA expression on human renal proximal tubular epithelial cells during hypoxia/reoxygenation. BMC Cell Biol..

[7] Lo P.H.Y., Urabe Y., Kumar V., Tanikawa C., Koike K., Kato N., Miki D., Chayama K., Kubo M., Nakamura Y. (2013). Identification of a functional variant in the MICA promoter which regulates MICA expression and increases HCV-related hepatocellular carcinoma risk. PLoS ONE.

[8] Steinle A., Li P., Morris D.L., Groh V., Lanier L.L., Strong R.K., Spies T. (2001). Interactions of human NKG2D with its ligands MICA, MICB, and homologs of the mouse RAE-1 protein family. Immunogenetics.

[9] Isernhagen A., Schilling D., Monecke S., Shah P., Elsner L., Walter L., Multhoff G., Dressel R. (2016). The MICA-129Met/Val dimorphism affects plasma membrane expression and shedding of the NKG2D ligand MICA. Immunogenetics.

[10] Isernhagen A., Malzahn D., Bickeböller H., Dressel R. (2016). Impact of the MICA-129Met/Val Dimorphism on NKG2D-Mediated Biological Functions and Disease Risks. Front. Immunol..

[11] Boukouaci W., Busson M., Peffault de Latour R., Rocha V., Suberbielle C., Bengoufa D., Dulphy N., Haas P., Scieux C., Amroun H. (2009). MICA-129 genotype, soluble MICA, and anti-MICA antibodies as biomarkers of chronic graft-versus-host disease. Blood.

[12] Isernhagen A., Malzahn D., Viktorova E., Elsner L., Monecke S., von Bonin F., Kilisch M., Wermuth J.M., Walther N., Balavarca Y. (2015). The MICA-129 dimorphism affects NKG2D signaling and outcome of hematopoietic stem cell transplantation. EMBO Mol. Med..

[13] Carapito R., Jung N., Kwemou M., Untrau M., Michel S., Pichot A., Giacometti G., Macquin C., Ilias W., Morlon A. (2016). Matching for the nonconventional MHC-I MICA gene significantly reduces the incidence of acute and chronic GVHD. Blood.

[14] Fuerst D., Neuchel C., Niederwieser D., Bunjes D., Gramatzki M., Wagner E., Wulf G., Glass B., Pfreundschuh M., Einsele H. (2016). Matching for the MICA-129 polymorphism is beneficial in unrelated hematopoietic stem cell transplantation. Blood.

[15] Askar M., Sobecks R., Wang T., Haagenson M., Majhail N., Madbouly A., Thomas D., Zhang A., Fleischhauer K., Hsu K. (2017). MHC Class I Chain-Related Gene A (MICA) Donor-Recipient Mismatches and MICA-129 Polymorphism in Unrelated Donor Hematopoietic Cell Transplantations Has No Impact on Outcomes in Acute Lymphoblastic Leukemia, Acute Myeloid Leukemia, or Myelodysplastic Syndrome: A center of international blood and marrow transplant research study. Biol. Blood Marrow Transplant..

[16] Cox S.T., Stephens H.A.F., Fernando R., Karasu A., Harber M., Howie A.J., Powis S., Zou Y., Stastny P., Madrigal J.A. (2011). Major histocompatibility complex class I-related chain A allele mismatching, antibodies, and rejection in renal transplantation. Hum. Immunol..

[17] Tonnerre P., Gerard N., Chatelais M., Poli C., Allard S., Cury S., Bressollette C., Cesbron-Gautier A., Charreau B. (2013). MICA variant promotes allosensitization after kidney transplantation. J. Am. Soc. Nephrol..

[18] Suárez-Alvarez B., López-Vázquez A., Díaz-Molina B., Bernardo-Rodríguez M.J., Alvarez-López R., Pascual D., Astudillo A., Martínez-Borra J., Lambert J.L., González S. (2006). The predictive value of soluble major histocompatibility complex class I chain-related molecule A (MICA) levels on heart allograft rejection. Transplantation.

[19] Baranwal A.K., Goswami S., Bhat D.K., Kaur G., Agarwal S.K., Mehra N.K. (2018). Soluble Major Histocompatibility Complex Class I related Chain A (sMICA) levels influence graft outcome following Renal Transplantation. Hum. Immunol..

[20] Ashiru O., López-Cobo S., Fernández-Messina L., Pontes-Quero S., Pandolfi R., Reyburn H.T., Valés-Gómez M. (2013). A GPI anchor explains the unique biological features of the common NKG2D-ligand allele MICA*008. Biochem. J..

[21] Seidel E., Le V.T.K., Bar-On Y., Tsukerman P., Enk J., Yamin R., Stein N., Schmiedel D., Oiknine Djian E., Weisblum Y. (2015). Dynamic co-evolution of host and pathogen: HCMV downregulates the prevalent allele MICA∗008 to escape elimination by NK cells. Cell Rep..

[22] Chalupny N.J., Rein-Weston A., Dosch S., Cosman D. (2006). Down-regulation of the NKG2D ligand MICA by the human cytomegalovirus glycoprotein UL142. Biochem. Biophys. Res. Commun..

[23] Ashiru O., Bennett N.J., Boyle L.H., Thomas M., Trowsdale J., Wills M.R. (2009). NKG2D ligand MICA is retained in the *cis*-Golgi apparatus by human cytomegalovirus protein UL142. J. Virol..

[24] Moenkemeyer M., Heiken H., Schmidt R.E., Witte T. (2009). Higher risk of cytomegalovirus reactivation in human immunodeficiency virus–1–infected patients homozygous for MICA5.1. Hum. Immunol..

[25] Cerboni C., Neri F., Casartelli N., Zingoni A., Cosman D., Rossi P., Santoni A., Doria M. (2007). Human immunodeficiency virus 1 Nef protein downmodulates the ligands of the activating receptor NKG2D and inhibits natural killer cell-mediated cytotoxicity. J. Gen. Virol..

[26] Goodier M.R., Jonjić S., Riley E.M., Juranić Lisnić V. (2018). CMV and natural killer cells: Shaping the response to vaccination. Eur. J. Immunol..

[27] Odom C.I., Gaston D.C., Markert J.M., Cassady K.A. (2012). Human herpesviridae methods of natural killer cell evasion. Adv. Virol..

[28] Racusen L.C., Solez K., Colvin R.B. (1999). The Banff 97 working classification of renal allograft pathology. Kidney Int..

[29] Nückel H., Switala M., Sellmann L., Horn P.A., Dürig J., Dührsen U., Küppers R., Grosse-Wilde H., Rebmann V. (2010). The prognostic significance of soluble NKG2D ligands in B-cell chronic lymphocytic leukemia. Leukemia.

