# Supplementary material for: A Valine Mismatch at Position 129 of MICA Is an Independent Predictor of Cytomegalovirus Infection and Acute Kidney Rejection in Simultaneous Pancreas–Kidney Transplantation Recipients
Source: Int J Mol Sci. 2018 Sep 4;19(9):2618. doi: 10.3390/ijms19092618 (PMC6164160; doi:10.3390/ijms19092618)
Supplement: Supplementary file 1 [file ijms-19-02618-s001.pdf]

International Journal of
Molecular Sciences

MDPI

Table S1. Primer sequences and PCR condition used for MICA rs1051792 genotyping.

PCR primers and conditions	Sequence (5′->3′)	Product size
**MICA1-F**	CAGGGAGGCATACCCCCTG	864 bp
**MICA1-R**	TCCGGGACCCCTGACCTG	
**MICA2-F***	GGGTCTGTGAGATCCATGA	127 bp
**MICA2-R***	TGAGCTCTGGAGGACTGGGGTA	
**PCR Steps**	Temperature (°C)-Duration	Cycles
**Initial denaturation**	95°C–2 min	1
**Denaturation**	95°C–30 sec	
**Primer annealing**	62.5°C–40 sec	40
**Extension**	72°C–1 min, 15 sec*	
**Final step**	72°C–5 min	1

The first PCR was prepared in a final volume of 30 μL: 1X Master mix (Ampliqon), 0.33μM of each MICA-1 primer and 1μL [50ng/μL] DNA. *Second PCR was prepared in final volume of 39.5 μL: 1X master mix, 0.33 μM of each MICA-2 primer and 0.5μL from first PCR product. Bp=base pairs. Min=minutes, Sec=seconds.

Table S2. Primer sequence and PCR condition used for MICA rs2596538A/G genotyping.

PCR primers and conditions	Sequence (5′->3′)	Product size
**MICA538F**	GTGAGTGCATGGGGTATAAGGC	339 bp
**MICA538R**	GTGCCAGCTCCAGCA AAGGAT	
**PCR Steps**	Temperature (°C)-Duration	Cycles
**Initial denaturation**	94°C–5 min	1
**Denaturation**	94°C–30 sec	
**Primer annealing**	56°C–30sec	32
**Extension**	72°C–1 min	
**Final step**	72°C–5 min	1

PCR was performed in a final volume of 25μL, 1X PCR Buffer–MgCl2 (Invitrogen), 2mM MgCl2 (Invitrogen), 0.4 μM of each primer, 0.5 mM dNTPmix (Thermo Fisher Scientific), 1Unit of Taq Platinum DNA (Invitrogen) and 1 μL DNA [50ng/μL]. Bp=base pairs. Min=minutes, Sec=seconds.
